# Genetic Variability of Wild Cherry (*Prunus avium* L.) Seed Stands in Slovenia as Revealed by Nuclear Microsatellite Loci

**DOI:** 10.1371/journal.pone.0041231

**Published:** 2012-07-20

**Authors:** Kristjan Jarni, Bart De Cuyper, Robert Brus

**Affiliations:** 1 Department of Forestry and Renewable Forest Resources, Biotechnical Faculty, University of Ljubljana, Ljubljana, Slovenia; 2 Department of Management and Sustainable Use, Research Institute for Nature and Forest, Geraardsbergen, Belgium; 3 Department of Forestry and Renewable Forest Resources, Biotechnical Faculty, University of Ljubljana, Ljubljana, Slovenia; United States Department of Agriculture, United States of America

## Abstract

Microsatellite markers were used to describe the genetic variability of four seed stands of wild cherry (*Prunus avium* L.). One hundred and thirty one individuals were genotyped at ten nuclear microsatellite loci. Total genetic diversity was high (*H*_E_ = 0.704), while differences between stands were small but significant (*F*_ST_ = 0.053, *G′*_ST_ = 0.234). There was a significant amount of clonal reproduction in one stand, with only 11 genotypes identified among 36 trees. One stand showed a significant excess (*F*_IS_ = −0.044) of heterozygosity, and one showed a deficit (*F*_IS_ = 0.044). Our results demonstrate the importance of taking into account the biological and genetic characteristics of species in forest management, especially when determining a new seed stand. The small genetic differences found between seed stands indicate that a large number of stands are not required. However, they should be carefully selected and should possess adequate genetic variability to ensure low relatedness between seed trees.

## Introduction

Tree regeneration in sustainable forest management requires high quality and genetically variable forest reproduction material (FRM) capable of achieving all of the objectives of future forest management. Genetic diversity of species is central for ensuring sustainability and ecosystem multi-functionality in the face of an unpredictable future, particularly in the context of global climate change. ‘Close to nature forestry’ in Central Europe primarily relies on natural regeneration; however, artificial regeneration is required in areas where natural regeneration is inhibited for various reasons. Most FRM used for artificial regeneration is sourced from seedlings that were grown from seeds collected in seed stands and seed orchards.

Wild cherry (*Prunus avium* L.) is a valuable broadleaved tree species that occurs naturally in western Eurasia and Northern Africa. It has a mostly scattered distribution and natural regeneration prevails. It is considered highly important from an economic (timber production) and environmental perspective [1]. In addition, a number of cultivars have been developed for fruit production. For these reasons, the species has been the subject of numerous genetic studies (e.g. [2], [3], [4], [5]). Due to the economic potential of its highly valuable wood, many European countries have developed intensive breeding programs, and much attention has also focused on the preservation of its forest genetic resources (e.g. [6], [7]).

Wild cherry is native in Slovenia and, as in most other countries, has a scattered distribution and normally regenerates naturally. The natural regeneration of wild cherry in Slovenia is generally poor, however, since the prevailing forestry system in Slovenia is a continuous cover forestry system that promotes the regeneration of species that are more shade tolerant. The regeneration is further hindered by game browsing and low competitiveness compared with other tree species (e.g. beech). However, wild cherry is a species with many desirable characteristics. Increasing its proportion and quality in existing deciduous and mixed forests would not only greatly increase the timber production value of these forests, but it could also provide other important functions. For example, it could be an important species for converting unnatural stands (e.g. spruce monocultures) to more natural structures.

Based on the assumption that natural selection has optimised populations to their local environment, European and national guidelines [8], [9], [10] advocate and encourage the use of high-quality local tree seed that is genetically and phenotypically adapted to the site. If seed harvesting is linked exclusively to seed stands, as is the case in Slovenia where there are no seed orchards, the selection of proper seed stands is crucial. Slovenia has ten registered seed stands of wild cherry. For minority tree species, the entire territory of Slovenia is considered a single provenance region, which means that the FRM collected in any seed stand can be used anywhere in Slovenia. The only condition is that the FRM be used in the same altitude belt. Due to irregular and weak fruiting and tall seed trees in existing seed stands, it is very difficult to secure a sufficient quantity of genetically variable FRM. In addition, the harvesting of seed from tall trees is very expensive.

Entomophily, zoochory, and generative reproduction with developed self-incompatibility mechanisms, along with vegetative propagation via root suckers, are the main generators of wild cherry's genetic composition in natural stands [3], [11], [12], [13], [14]. In addition to natural influences, the genetic composition of wild cherry stands is affected by forest management practices [11], [14], an example being ‘close to nature forestry’, where crop trees are selected through tending treatments. All of these factors must also be taken into account when selecting seed stands and harvesting the seed itself. Moreover, knowledge of the biological and genetic characteristics of wild cherry is necessary for intensifying its breeding, utilising its economic potential, and protecting its gene pool. The genetic population characteristics of wild cherry in Slovenia have not yet been studied. The only exception is a sample of 10 trees from a population on Mt. Slavnik, which was included in the study of Guarino et al. [15]. This study showed a low genetic differentiation between this population and populations from Croatia and northern Italy and somewhat greater in relation to two populations from the Apennines. However, in this paper we focus on the genetic variability of four wild cherry seed stands. The stands were genetically analysed with ten nuclear microsatellites i) to examine their genetic variability and ii) to determine whether such genetic variability is sufficient to allow their further use as a source of genetically variable FRM. This insight into the genetic diversity and structure of the stands could also be important for conservation activities.

## Materials and Methods

### Sampling

Four natural stands of wild cherry were sampled in Slovenia. Material was collected from at least 30 adult trees at each site. One of the stands (stand C) is managed forest while the others are unmanaged (Table 1). In addition, stands A and B are the result of natural reforestation that recently occurred in a large open area. Nevertheless, all stands are registered as official seed stands where seeds are collected for forest regeneration. Trees that were at least 50 m apart were randomly chosen in stands A, B, and C, while sampling in stand D was slightly modified. Stand D is located at the bottom of a ca. 70 m wide depression and is geographically restricted by steep slopes on all four sides. We sampled ca. 15% of all cherry trees in this small and dense stand where all trees are actually neighbours. Young leaves from each tree were collected and put directly on silica gel until DNA extraction. No specific permits were required for this field study. The Slovenia Forest Act declares that the forest in Slovenia is a public space where anyone can enter and collect forest products, regardless of ownership, except timber that belongs to the forest owner. In addition, the field study did not involve endangered or protected species.

**Table 1:** Main characteristics of the seed stands.

Stand	Latitude	Longitude	No. of trees	Research area (ha)	Treatment	Altitude#
A	45°35′20″N	14°13′57″E	32	55	unmanaged	420–470
B	45°58′10″N	14°16′39″E	32	16	unmanaged	320–380
C	46°23′03″N	16°01′55″E	31	11	managed	220–310
D	45°46′25″N	13°59′31″E	36	0.5	unmanaged	360–370

# = above mean sea level.

### Microsatellite analysis

Total DNA was extracted with the DNeasy® Plant Mini Kit (Qiagen) from approximately 20 mg of dried leaves ground in an automatic grinding mill (Retsch MM200). All individuals were genotyped at ten nuclear microsatellite loci. Two multiplex polymerase chain reactions (PCR) were carried out [16] as well as three simplex PCRs (EMPa15, UDP98-412 and PceGA34). The multiplex reactions were carried out using the following combinations of primers: Multiplex–A: EMPaS10, EMPaS12, and EMPaS14; Multiplex–B: EMPa4, EMPa5, EMPaS2, EMPaS6. The forward primers were labelled with fluorescent dyes FAM, HEX, or NED (Applied Biosystems). The Multiplex PCRs were performed according to the protocol of Vaughan and Russell [16]. The simplex PCR for the locus EMPa15 followed the protocol of Clarke and Tobutt [17]. The conditions for the remaining two simplex PCRs were: 94°C for 2 min. followed by 35 cycles of 94°C for 30 s, Ta°C for 45 s, and 72°C for 60 s with an elongation step of 72°C for 5 min. PCR products were genotyped with a capillary sequencer (SpectruMedix model SCE 960) and scored using GenoSpectrum software.

For each of the microsatellite loci, we ascertained the total number of alleles, the range of allele sizes, and the gene diversity *H*_E_ to assess overall polymorphisms. Additionally, for averages over loci we computed the average number of observed alleles *A*, the observed heterozygosity *H*_O_, the expected heterozygosity *H*_E_, and Wright's inbreeding coefficient *F*_IS_ for each of the four seed stands. The software ARLEQUIN [18] was applied for the analysis mentioned above while conformation of genotypic frequencies to the Hardy-Weinberg equilibrium was tested with the program GENEPOP version 4.0 [19]. Differences between stands were analysed based on allele identity with the statistic *F*_ST_ and standardized values *G′*_ST_ calculated according to Hedrick [20]. The private allele method published in Barton and Slatkin [21] was used to estimate the number of reproductively successful migrants per generation (*Nm*).

Spatial genetic structure was assessed according to Rousset [22] by testing the effect of distance on genetic divergence. The relationship between genetic distance, expressed as *F*_ST_/(*1*−*F*_ST_) and the natural logarithm of the geographic distance was analysed with Spearman's rank correlation and its significance was assessed through the Mantel test with the software program GENEPOP version 4.0 [19].

## Results

### Allelic diversity of microsatellite loci

The results showed very high polymorphism at locus EMPaS10; different alleles were distinguished by only one base, resulting in very difficult and therefore unreliable scoring. For this reason we excluded this locus from further analysis. Among the remaining nine polymorphic loci we detected a total of 71 SSR alleles in 131 individuals, with the number of alleles per locus ranging from 3 to 14 (Table 2). In stand D a few clonal colonies were found, as we detected only 11 different genotypes in the sample size of 36 trees. On the basis of the allele frequencies of the population, the probability that two unrelated individuals share an identical nine-locus DNA profile by chance is less than 0.024%. That we are dealing with clones can be inferred from the fact that all individuals that share the same genotype are neighbours. Although the genetic structure of wild cherry populations is affected by both generative and vegetative propagation, we decided to include only one representative per clone group in the statistical analysis.

**Table 2:** Allelic diversity of the nuclear microsatellite loci used in the analysis.

Locus	N	*A*	*A*_e_	*H*_E_	*F*_IS_	*F*_ST_	Hardy–Weinberg equilibrium test			
							A	B	C	D
EMPaS02	104	7	3.205	0.737	0.094 n.s.	0.049**	n.s.	n.s.	n.s.	n.s.
EMPa004	105	6	2.634	0.694	0.067 n.s.	0.136***	n.s.	n.s.	n.s.	**
EMPa005	104	8	3.079	0.701	−0.052 n.s.	0.029*	n.s.	n.s.	n.s.	n.s.
EMPaS06	103	8	3.899	0.780	−0.046 n.s.	0.033*	n.s.	n.s.	n.s.	**
EMPaS12	106	6	3.155	0.729	−0.081 n.s.	0.058***	n.s.	**	*	*
EMPaS14	106	3	1.703	0.439	0.067 n.s.	0.045*	n.s.	n.s.	n.s.	n.s.
EMPa015	101	13	3.643	0.759	0.064 n.s.	0.035*	n.s.	n.s.	n.s.	n.s.
UDP98-412	91	6	3.613	0.747	−0.025 n.s.	0.014 n.s.	n.s.	n.s.	n.s.	n.s.
PceGA34	98	14	4.101	0.796	0.015 n.s.	0.034*	n.s.	n.s.	n.s.	n.s.
Multilocus#				0.704	0.012	0.053				
Permutation test					P>0.05	P<0.001				

N = number of trees; *A* = total number of alleles; *A*_e_ = effective number of alleles; *H*_E_ = gene diversity (corrected for sample size [23]); *F*_IS_ = Wright's inbreeding coefficient; *F*_ST_ = relative differentiation based on allele identity,

# = multilocus estimates without locus UDP98-412, (n.s. P>0.05;

* 0.01<P<0.05;

** 0.001<P<0.01).

The analysis showed, notwithstanding three separate trials, that locus UDP98-412 did not give successful amplification in a large proportion of individuals in stands C and D (35% and 33% respectively). These features could be associated with the presence of null alleles at high frequency or unreliable amplification reactions. Reliable answers could have been obtained from the analyses of offspring from controlled crossing. However, Table 2 shows the basic characteristics of all studied loci, including locus UDP98-412, which was excluded from further statistical analysis despite its small contribution to the final results and conclusions (data not shown).

Overall gene diversity (*H*_E_) was very similar across loci with the exception of locus EMPaS14, which showed more than 40% less diversity than the other loci. Moderate variation in *F*_ST_ (ranging from 0.014 to 0.136) and higher variation in *F*_IS_ (ranging from −0.081 to 0.094) were observed among loci (Table 2). The average expected number of alleles in a locus in each stand (*A*_e_) varied between 1.703 for the least diverse locus EMPaS14 and 4.101 for the most various locus PceGA34. This number (*A*_e_), which refers to an ideal Wright-Fisher population, is less than half the number of alleles of the actual stands (comparison between *A*_e_ and *A*) except for the most various loci EMPa015 and PceGA34, where the effective number (*A*_e_) amounted to only one-third of the actual number of alleles (*A*). For the majority of loci in individual seed stands, we found no noticeable deviation from Hardy-Weinberg equilibrium when only sexually derived individuals were considered (Table 2). The exceptions were locus EMPaS12, which showed departures from HWE due to an excess of heterozygotes in two seed stands and deficit in one, locus EMPaS06, which showed excess of heterozygotes in stand D, and locus EMPa004, which showed deficit of heterozygotes in the same stand.

### Genetic variation within stands

Moderate genetic polymorphism was found within stands; on average approximately five alleles per locus were detected (*A* = 5.66±1.1 SD) (Table 3). The probability that any two alleles at a single locus, chosen at random from the stands, were different from each other was 66% (*H*_E_ = 0.658). The comparison between observed heterozygosity (*H*_O_ = 0.656) and expected heterozygosity (*H*_E_) showed that stands were on average slightly, but not significantly, inbred, with a mean inbreeding coefficient *F*_IS_ = 0.001 (±0.048 SD). Two stands indicated an overall significant deviation from the Hardy-Weinberg genotypic expectation: stand D showed an excess of heterozygosity and stand B showed a deficit.

**Table 3:** Statistics of genetic variation within stands at eight microsatellite loci.

Stand	N	*A*	*H*_O_	*H*_E_	*F*_IS_#
A	32	5.875	0.652	0.680	0.041
B	32	6.750	0.661	0.692	0.044*
C	31	5.875	0.717	0.692	−0.036
D	11	4.125	0.593	0.568	−0.044**
Overall men		5.656	0.656	0.658	0.001
Standard deviation		1.101	0.051	0.060	0.048

N = number of trees in the stand, *A* = average number of alleles per locus, *H*_O_ = observed heterozygosity, *H*_E_ = average expected heterozygosity, *F*_IS_ = the deficiency or excess of average heterozygosity,

# = exact test of departure from Hardy–Weinberg genotypic proportions (* 0.01<P<0.05; ** 0.001<P<0.01; all other values, not significant).

### Genetic variation between stands

The total genetic diversity of wild cherry from the four stands was high (*H*_E_ = 0.704±0.11 SD), while differences between stands were low but significant (*F*_ST_ = 0.053, *G′*_ST_ = 0.234). Consequently, the estimated number of migrants was high (*Nm* = 4.2). However, the largest differences were found between stand D and stands B and C, while the differences between the others were smaller (Table 4). Correlation analysis indicated that pairwise genetic similarity between the four seed stands, expressed as *F*_ST_/(*1*−*F*_ST_), was not significantly related to the logarithm of the geographical distance between stands (R^2^ = 0.007, P>0.05).

**Table 4:** Stand pairwise *F*_ST_ and *G′*_ST_ in the brackets.

Stand	A	B	C
B	0.032 (0.086)		
C	0.053 (0.146)	0.043 (0.122)	
D	0.054 (0.130)	0.097 (0.239)	0.085 (0.209)

## Discussion

Based on nuclear microsatellite markers, the genetic variability in the four natural stands of wild cherry is high. The number of alleles per locus found in this study (Table 2) is comparable to that obtained in other studies, such as a study on wild cherry from Kent (UK) [14]. In addition, like Vaughan et al. [14], we found that one of the least variable loci was EMPa14 and the most variable was PceGA34. However, it seems that the pattern of genetic variability in wild cherry shares the general pattern of variability characteristic of long-lived, outcrossing tree species (e.g. [12], [24], [25], [26]), which is characterised by high genetic diversity (*H*_E_ = 0.704), a low level of inbreeding within populations (*F*_IS_ = 0.012), and low differentiation among populations (*F*_ST_ = 0.053, *G′*_ST_ = 0.234).

For the majority of loci in individual seed stands, we found no noticeable deviation from Hardy-Weinberg equilibrium (Table 2). On the seed stand level, there was real deviation from HWE in stand D, which showed an excess of heterozygotes, and stand B, which showed a deficit (Table 3). The latter could be explained by slight inbreeding. Although this would also be expected in stand D due to its very small and restricted size, the results showed just the opposite. Stand D contained 4.4% more heterozygotes than would be expected. One of the reasons for this could be that wild cherry is self-incompatible [27]. This mechanism is considered to be one of the main reasons for the excess of heterozygotes observed in the species *Sorbus torminalis* [28]. For this reason, one would always expect an excess of heterozygotes in wild cherry, but this was not the case in this study or in the studies of other authors (e.g. [14]). However, heterozygote excess in wild cherry has also been reported by others [29], [13]. The latter study indicates clonality as the most likely reason for the heterozygote excess, but in our case this could not have been the cause since only one representative per clone group was analysed in a particular stand. Another possible reason for heterozygotes excess, although unconfirmed by the survey, could be the impact of small population size. In such populations, a low proportion of homozygotes in the offspring may occur when only a few breeders participate in mating, and the allele frequency between mating types can differ by chance alone [13]. For stand D this could mean that the parental population was comprised of a small number of genetically different individuals. However, we do not have any information to confirm this. The reason may also lie in the methodology itself. Namely, by including only one representative per clone group in the analysis, the sample size was greatly reduced, resulting in operative error and less reliable conclusions. In addition, by excluding ramets, we may have violated the random mating rule, which is one of the preconditions for the Hardy-Weinberg equilibrium law. Although this is unlikely, with the probability being less than 0.024%, we may have omitted some trees from the analysis that had identical alleles by chance.

Previous genetic studies have shown that the genetic composition of wild cherry populations is significantly influenced by the type of reproduction [3], [12], [14]. As a species that frequently reproduces vegetatively, its natural regeneration could also lead to a significant reduction in genetic variability [11], [14]. Our study showed that seed stand D had the lowest genetic variability. We detected only 11 different genotypes in the sample of 36 trees, indicating that a large proportion of the stand had a vegetative origin. If we add to this the self-incompatibility mechanism in the stands where individual trees are actually clones [3], [28], we can say that stand D is particularly inappropriate to perform the role and mission of a seed stand. Although it has been suggested that the problem of narrow kinship among trees can be avoided by seed gathering from the trees that are spaced at least 100 m apart [14], this was impossible to accomplish in this case because of the small size of the stand. However, a visible feature that indicates the vegetative origin of wild cherry is its high density in the stands and short distances between individual trees. Adulthood and the size of individual trees should not mislead us about their origin. Although this was not an issue in this study, field observations confirmed high phenotypic similarities between individual ramets, such as stem form, spiral growth, and frost crack formation.

This study has shown that the size of the sampling area did not significantly affect the observed variability in the populations, with the exception of stand D, where the small size of the stand prevented the sampling of unrelated trees. A similar finding on the relationship between population size and variability was also reported in a study of *Sorbus torminalis*, which showed that small populations did not harbour less genetic diversity [28]. In this research, the highest genetic variability was detected in stand C (Table 3), where the sampling was performed in an area of 11 hectares, while the sampling areas of stands A and B were substantially larger. It seems that the regular tending measures in stand C, which were applied through the whole stand development process, did not affect the neutral genetic variability in the stand. On the basis of nuclear microsatellites, Vaughan et al. [14] noted that measures such as thinning can maintain or even increase the spatial genetic structure (SGS) over smaller distances in wild cherry. This is supported by the fact that many economically important traits are under strong genetic control (e.g. [30], [31], [32], [33]). Vaughan et al. [14] suggested that the removal of undesirable phenotypes may lead to the homogenization and promotion of family groups sharing desirable traits that result in increased SGS. The latter should be considered during the design of seed stands. The design of a seed stand should not only consider the superiority of individual trees, but also their spacing across the stand. Namely, a regular distribution of trees prevents the promotion of whole families which could be phenotypically superior, but, because of their relatedness, less genetically variable. This could explain the slightly higher genetic variability in stand C, where the trees are more or less evenly distributed across the stand, possibly indicating that they arise from a larger pool of parents. Through regular tending, managers probably nurtured some trees with inferior genotypes to become large, valuable trees. This is possible because high phenotypic plasticity could blur the genetically weak determined traits, which are often quantitative traits (e.g. diameter, height, vitality) that are important for forest management. Without testing the progeny, we cannot distinguish them from the trees with good genotypes, which means that they will participate in regeneration processes in the future and will reduce the genetic gain. On the other hand, stands A and B are a result of natural reforestation that recently occurred in a large open area. One of the plausible explanations for their slightly lower variability could lie in the lower diversity of parental trees, both in their number and genetic constitution. These two stands are not characterised by ‘superior’ trees, but are listed as seed stands due to the lack of these stands in Slovenia. Nevertheless, the convenience of harvesting in natural stands should only override the ‘phenotypic superiority’ of the trees in extreme cases such as ours, in which there is no other choice.

## Conclusions

The total genetic diversity of wild cherry from the four seed stands was high. Although the sampling method implied some heterogeneity, the differences between these stands were small. This pattern of variation implies that the creation of a small number of seed stands is sufficient, but that stands must be spread out over a large enough area to ensure seeds are collected from unrelated trees. The problem of clonal origin was found in stand D, where a large number of trees are of vegetative origin. Such a stand cannot perform the functions of a seed stand and should be removed from the list of registered seed stands. The highest genetic variability was detected in stand C, possibly the result of regular tending in this stand. We found that three out of four studied seed stands fully meet the conditions that a seed stand should fulfil with respect to genetic variation. These findings support the decision that Slovenia is one provenance unit for wild cherry.
